# Integration of metagenomic and metabolomic insights into the effects of microcystin-LR on intestinal microbiota of *Litopenaeus vannamei*

**DOI:** 10.3389/fmicb.2022.994188

**Published:** 2022-09-23

**Authors:** Yafei Duan, Yifu Xing, Shimin Zeng, Xueming Dan, Zequan Mo, Jiasong Zhang, Yanwei Li

**Affiliations:** ^1^Key Laboratory of South China Sea Fishery Resources Exploitation and Utilization, Ministry of Agriculture and Rural Affairs, Guangdong Provincial Key Laboratory of Fishery Ecology and Environment, South China Sea Fisheries Research Institute, Chinese Academy of Fishery Sciences, Guangzhou, China; ^2^Key Laboratory of Efficient Utilization and Processing of Marine Fishery Resources of Hainan Province, Sanya Tropical Fisheries Research Institute, Sanya, China; ^3^University Joint Laboratory of Guangdong Province, Hong Kong and Macao Region on Marine Bioresource Conservation and Exploitation, Guangdong Laboratory for Lingnan Modern Agriculture, College of Marine Sciences, South China Agricultural University, Guangzhou, China

**Keywords:** shrimp, microcystin-LR, intestinal microbial, gene function, metabolites

## Abstract

Microcystin-LR (MC-LR) is a hazardous substance that threaten the health of aquatic animals. Intestinal microbes and their metabolites can interact with hosts to influence physiological homeostasis. In this study, the shrimp *Litopenaeus vannamei* were exposed to 1.0 μg/l MC-LR for 72 h, and the toxic effects of MC-LR on the intestinal microbial metagenomic and metabolomic responses of the shrimp were investigated. The results showed that MC-LR stress altered the gene functions of intestinal microbial, including ABC transporter, sulfur metabolism and riboflavin (VB2) metabolism, and induced a significant increase of eight carbohydrate metabolism enzymes. Alternatively, intestinal metabolic phenotypes were also altered, especially ABC transporters, protein digestion and absorption, and the biosynthesis and metabolism of amino acid. Furthermore, based on the integration of intestinal microbial metagenomic and metabolome, four bacteria species (*Demequina globuliformis*, *Demequina* sp. *NBRC 110055*, *Sphingomonas taxi* and *Sphingomonas* sp. *RIT328*) and three metabolites (yangonin, α-hederin and soyasaponin ii) biomarkers were identified. Overall, our study provides new insights into the effects of MC-LR on the intestinal microbial functions of *L. vannamei*.

## Introduction

The Pacific white shrimp *Litopenaeus vannamei* is an important economic aquatic animal species. Harmful cyanobacteria are the dominant phytoplankton in shrimp ponds. With the increase of eutrophication level in the rearing water, cyanobacteria are easy to multiply, which not only pollutes the water quality, but also releases a large amount of cyanotoxins, threatening the health and food safety of shrimp (Magalhães et al., 2003; Pham and Utsumi, 2018). Microcystins (MCs) are one of the most common and highly toxic cyanotoxins, the concentration of which in cyanobacterial bloom ponds can reach 0.1–10 μg/l (Ye et al., 2014; Li et al., 2016). Microcystin-LR (MC-LR) is considered to be the most common and widely studied type of MCs, which are encountered in freshwater systems, estuarine and marine environments (Anderson et al., 1993; Ramos et al., 2005; Miller et al., 2010; Preece et al., 2017). It was reported that the MCs concentration of *L. vannamei* ponds was 0.53–2.25 μg/l at the initial stage, and reached 1.79–2.25 μg/l after 80 days of culture (Wang, 2018); the MC-LR content of *L. vannamei* ponds with dead individuals was as high as 45 μg/l (Zimba et al., 2006). MC-LR stress can cause immune disorder and induce disease susceptibility in shrimp (Chen et al., 2016, 2017; Yuan et al., 2016; Li et al., 2019a; Duan et al., 2020). Unfortunately, effective measures to prevent MC-LR stress in shrimp are still lacking.

The regulation of intestinal microecology has been proved to be beneficial to the prevention of shrimp diseases. The shrimp intestine inhabits a large microbial community including both beneficial and harmful bacteria, which can interact with their host. A stable intestinal microbiota is beneficial to the host health by participating in substance metabolism, promoting nutrient digestion and absorption, and acting as a biological barrier (Holt et al., 2021). However, intestinal microbiota unbalance will affect the host immune and metabolic homeostasis, and then induce the occurrence of diseases (Li et al., 2019b). Studies have shown that MC-LR stress can lead to intestinal microbiota composition variations and immune dysfunction in shrimp (Duan et al., 2020). Ten and forty microgram per liter MC-LR can induce a decrease in intestinal microbiota diversity and a change in community composition of *Procambarus clarkii* (Zhang et al., 2020b). Similar phenomena have been observed in mice (Chen et al., 2015; Zhang et al., 2016, 2020a; Zhuang et al., 2021) and zebrafish (Li et al., 2019a). Additionally, MC-LR stress can also alter the functional gene profiles of intestinal microbiota in rats, including chitin, starch and limonene metabolism (Lin et al., 2015). But the effects of MC-LR exposure on the intestinal microbial function of *L. vannamei* are absence.

Therefore, in this study, we aimed to explore the effects of MC-LR on the gene function and metabolic profile of intestinal microbial in *L. vannamei* using an integrated metagenomic and metabolomic approach. Our main goals were to reveal: (1) the changes of functional genes in intestinal microbial of MC-LR-stressed *L. vannamei*; (2) the metabolic characteristics in the intestines of MC-LR-stressed *L. vannamei*; (3) the changes of the major pathways and sensitive biomarkers that were closely associated with MC-LR stress. The results of this study are helpful to understand the response mechanism of intestinal microbial of *L. vannamei* to MC-LR stress.

## Materials and methods

### Shrimp and rearing conditions

Healthy *L. vannamei* juveniles with average body weights of 1.2 ± 0.2 g, were randomly collected from a local shrimp pond in Shenzhen, China. The shrimp were acclimated in 100 l experimental tanks for 7 days. Each tank was filled with 60 l of filtered seawater with adequate safety for shrimp and aerated continuously (salinity 30, temperature 30°C ± 0.2, pH 8.1 ± 0.1). The shrimp were fed commercial feed with 40% crude protein (Haida Feed, China), and the filtered seawater from the same source was renewed daily.

### Stress exposure and sample collection

MC-LR was purchased from Taiwan algal science Inc., the purity ≥95%. After acclimation, the shrimp were randomly divided into two groups: the control (CK), and the MC groups. Each group consisted of six replicate 100 l tanks that raised 40 shrimp in 60 l seawater per group. According to the report on the MC-LR toxicity in *L. vannamei*, 0.5–3.0 μg/l MC-LR can affect the immunity of *L. vannamei* (Chen et al., 2017b). In order to explore the toxicity of relatively low dose of MC-LR to *L. vannamei*, the concentration of MC-LR in this study was set as 1.0 μg/l, which was 1 folds of the WHO’s maximum standard for water (1.0 μg/l) (WHO, 2011). The CK group was normal seawater without MC-LR detection. The rearing water of the MC group contained 1.0 μg/l MC-LR, which were verified analytically. The water of each tank was all replaced every 24 h, and the MC-LR was re-added in the corresponding concentration. The culture conditions of the exposed stage were consistent with those of the acclimation stage.

After 72 h of MC-LR exposure, the intact intestines of 30 individual shrimps from each tank were collected. To reduce the impact of interindividual differences, the 30 intact intestines were homogenized and bisected into one metagenomic sample and one metabolome sample. Overall, six metagenomic samples and six metabolomic samples were collected from each group.

### Intestinal microbial metagenomic analysis

The genomic DNA was extracted using CTAB/SDS method and detected by 1.0% agarose gel electrophoresis. After DNA fragmentation, the Illumina NEBNext^®^ Ultra™ DNA Library Prep Kit (NEB, USA) was used to construct the sequencing library according to the manufacturer’s instructions. The libraries were sequenced on Illumina Novoseq platform. The obtained raw reads were processed to obtain high-quality clean data using Readfq software (V8). Then the host gene contamination was removed according to the genome of *L. vannamei* using Bowtie2 software (V2.2.4). The Scaftigs were obtained by SOAPdenovo software (V2.04). MetaGeneMark (V2.10) was used to predict the ORF of Scaftigs, and CD-HIT software (V4.5.8) was used to remove the redundancy and obtain the initial gene catalogue. Bowtie2 software (V2.2.4) was used to compare with clean data of each sample to obtain the final gene set for subsequent analysis. According to the gene set, the gene abundance information in each sample was counted, and the gene number difference between groups and the Venn diagram were analyzed. DIAMOND software (V0.9.9.110) was used to align Unigenes with non-redundant (NR) database, annotate species information, and analyze non-metric multidimensional scaling (NMDS) dimensionality reduction at different classification levels using R Vegan software package (V2.15.3). DIAMOND software (V0.9.9.110) was used to annotate Unigenes with Kyoto Encyclopedia of Genes and Genomes (KEGG), evolutionary genealogy of genes: Non-supervised Orthologous Groups (eggNOG), and carbohydrate active enzyme (CAZyme) databases, and the parameter was set to *E*-value ≤1E-5. The functional differences between the groups were analyzed by Metastat software. All the raw data of intestinal microbiota has been submitted to the Sequence Read Archive (SRA) database (accession: PRJNA862309).

### Intestinal metabolomics analysis

The samples were thawed slowly at 4°C, and homogenized with 200 μl of sterile water. The homogenized solution was mixed with 800 μl methanol/acetonitrile (1: 1, v/v) for metabolite extraction. The mixture was ultrasonicated at 4°C for 30 min, stood at–20°C for 10 min, and then centrifuged at 14,000*g*, 4°C for 20 min. The supernatant was vacuum dried and redissolved in 100 μl acetonitrile solution (acetonitrile:water = 1:1, v/v) for liquid chromatography-mass spectrometry (LC–MS) analysis.

Liquid chromatography was performed in an UHPLC (Agilent 1290 Infinity LC) coupled to a quadrupole time-of-flight (AB Sciex TripleTOF 6600). HILIC separation were analyzed using a ACQUIY UPLC BEH column (2.1 mm × 100 mm, 1.7 μm) (Waters, Ireland). Mass spectrometry was accomplished in an AB Triple TOF 6600 mass spectrometer to collect the primary and secondary spectra of the samples. The positive and negative ionization modes of electrospray ionization (ESI) were used for detection, respectively.

The raw MS data (wiff.scan files) were converted to MzXML files using ProteoWizard MSConvert before importing into freely available XCMS software. All the data were determined using total ion chromatogram (TIC) and quality control (QC). After sum-normalization, compound identification of metabolites was performed by comparing of accuracy m/z value (<25 ppm), and MS/MS spectra with an in-house database (Shanghai Applied Protein Technology) established with available authentic standards (Luo et al., 2017; Gu et al., 2018). All the metabolites were classified according to their chemical taxonomy. The variation trend of metabolite expression in each group was analyzed using fuzzy c-means (FCM) algorithm of Mfuzz packet. The visual volcano plot of the differential metabolites (DMs) was analyzed with an standard of fold-change (FC) > 1.5 or FC < 0.67, and *p* < 0.05. The metabolomic data were multiple compared with orthogonal partial least-squares discriminant analysis (OPLS-DA) using R package (ropls). The robustness of the model was evaluated by 7-fold cross-validation and response permutation test. The DMs of MC vs. CK were identified with an standard of OPLS-DA VIP > 1 and *p* < 0.05. The KEGG pathway of all the DMs was performed using KEGG software.^1^ The metabolite markers with significant changes were further screened and analyzed.

### Integrated analysis of intestinal microbial metagenomic and metabolomic

The KEGG pathways of differential genes and metabolites of intestinal bacteria were analyzed, and the pathway attribution of them were integrated. Based on the standardized data of scale, the spearman correlation coefficient between the differential bacteria and metabolites with linear discriminant analysis (LDA) > 2 were analyzed using R package psych software. The multilevel regulatory relationship among bacteria species, functional genes and metabolites was analyzed, and Sankey diagram was conducted using R package plotly software.

## Results

### Genes profile and functional changes of intestinal microbiota

**Figure 1:**
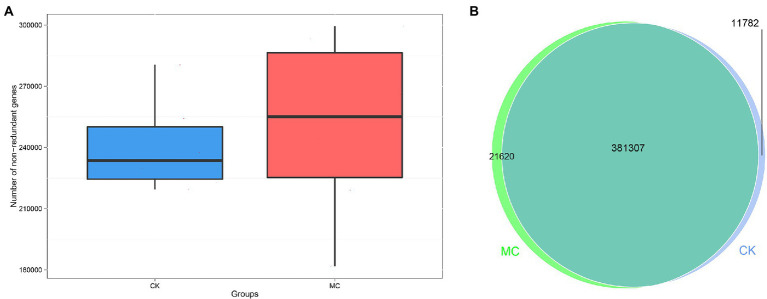
Gene numbers analysis of intestinal microbial of *L. vannamei* after MC-LR exposure. **(A)** Comparison of non-redundant genes number between the CK and MC groups. **(B)** Venn diagram of gene numbers between the CK and MC groups.

A total of 75,600 clean reads were obtained from 12 intestinal microbial samples in the two groups by metagenomic sequencing, with an average of 6,300 reads per sample; the effective were 96.63–99.75% (Supplementary Tables S1–S3). A total of 2,947,876 genes were obtained, and the number of genes in the MC group was more than that in the CK group (Figure 1A). Based on Venn analysis, 381,307 genes were co-owned by the two groups, and the unique genes of the MC group was higher than that of the CK group (Figure 1B).

**Figure 2:**
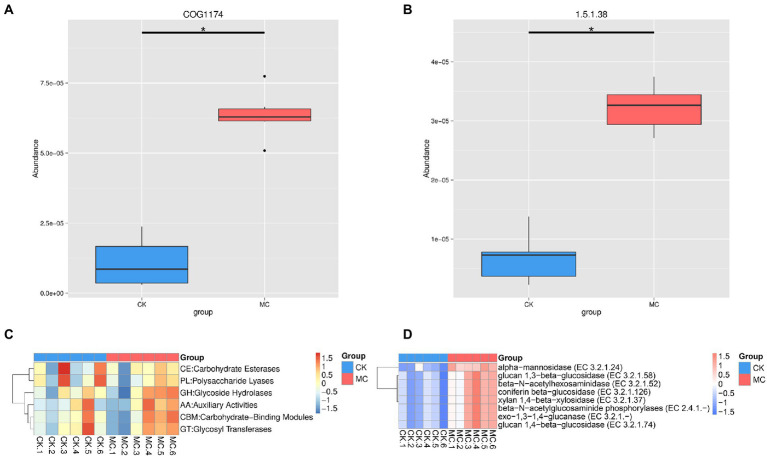
Significantly altered gene function of intestinal microbial of *L. vannamei* after MC-LR exposure. **(A)** eggNOG function. **(B)** KEGG function. **(C)** CAZy categories. **(D)** CAZy species. * represents significant difference between the CK and MC groups (*q* < 0.05).

Based on the eggNOG analysis, the highest functional categories was “amino acid transport and metabolism” followed by “energy production and conversion” “inorganic ion transport and metabolism” etc. (Supplementary Figure S1). At the level 2, the top categories were “ABC transporter” followed by “transcriptional regulator” “membrane” “histidine kinase” and “dehydrogenase” (Supplementary Figure S2). Compared with the CK group, “ABC transporter (COG1174)” was significantly increased in the MC group (Figure 2A). The KEGG analysis of all the functional genes was also performed, which were mainly “carbohydrate metabolism” “amino acid metabolism” “membrane transport” “translation” and “metabolism of cofactors and vitamins” (Supplementary Figures S3–S4). Compared with the CK group, the “KO_EC 1.5.1.38” pathway encoding “sulfur metabolism” and “riboflavin metabolism” was significantly increased in the MC group (Figure 2B).

### Altered CAZyme of intestinal microbiota

The CAZyme of intestinal microbe of the shrimp were further analyzed. The highest CAZyme taxa was glycoside hydrolases (GH), followed by glycosyltransferases (GT), carbohydrate-binding modules (CBM), carbohydrate esterases (CE), auxiliary activities (AA), and polysaccharide lyases (PL) (Supplementary Figure S5; Figure 2C). Compared with the CK group, the abundance of eight enzymes was significantly increased in the MC group, including α-mannosidase, glucan 1,3-β-glucosidase, glucan 1,4-β-glucosidase, coniferin β-glucosidase, β-N-acetylhexosaminidase, β-N-acetylglucosaminide phosphorylases, xylan 1,4-β-xylosidase, and exo-1,3-1,4-glucanase (Figure 2D).

### Changes in the composition of intestinal bacterial species

**Figure 3:**
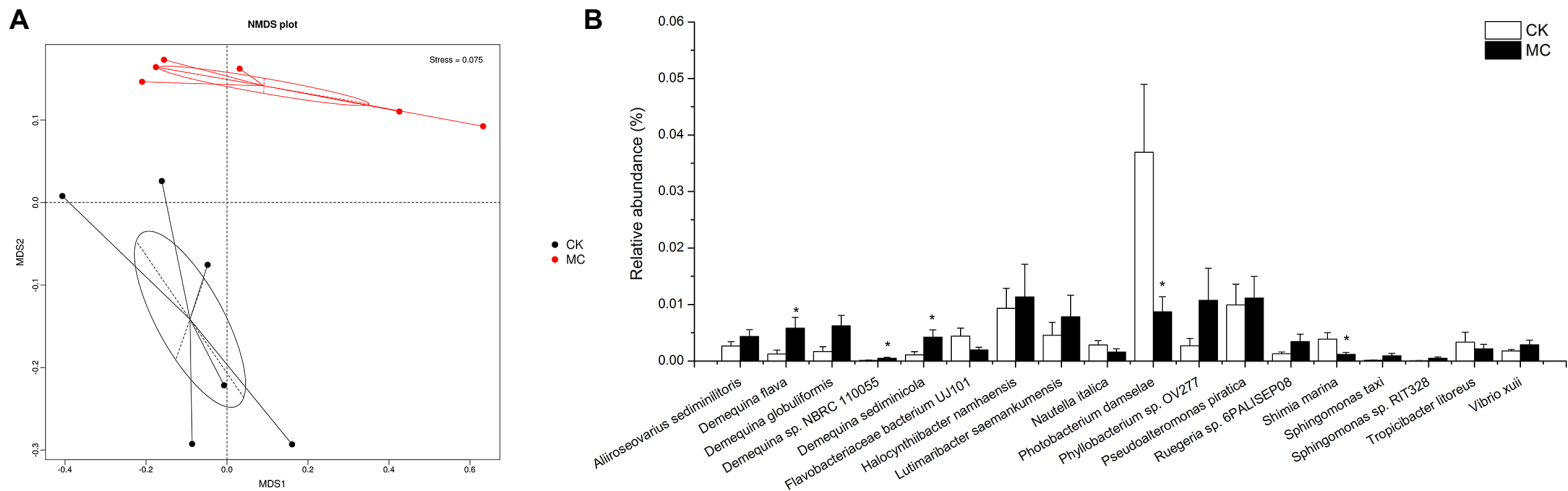
Comparison of intestinal bacteria species of *L. vannamei* after MC-LR exposure. **(A)** NMDS analysis. **(B)** The relative abundance of bacteria species. * Indicates significant differences between the two groups (*p* < 0.05).

Changes in intestinal microflora of the shrimp were analyzed. NMDS plot showed a clear visual separation in intestinal microbial composition between the CK and MC groups (Figure 3A). Several relatively high abundance bacterial species fluctuated in response to MC-LR exposure (Figure 3B). For example, the relative abundances of *Demequina flava*, *Demequina* sp. *NBRC 110055*, and *Demequina sediminicola* were significantly increased, while that of *Photobacterium damselae* and *Shimia marina* were significantly decreased. Additionally, *Demequina globuliformis*, *Phyllobacterium* sp. *OV277*, *Ruegeria* sp. *6PALISEP08*, *Sphingomonas taxi*, *Sphingomonas* sp. *RIT328*, and *Vibrio xuii* were increased, while *Flavobacteriaceae bacterium UJ101* and *Nautella italica* were decreased, but the differences were not significant.

### Screening and functional analysis of intestinal metabolites

**Figure 4:**
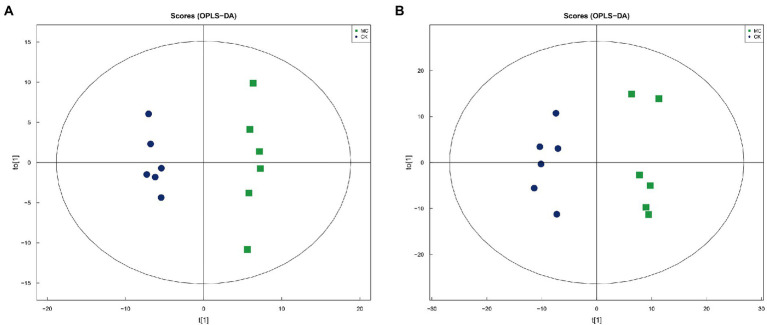
OPLS-DA analysis of intestinal metabolites of *L*. vannamei after MC-LR exposure. **(A)** Positive ion mode. **(B)** Negative ion mode

A total of 2,052 metabolites were identified, including 1,213 positive ion mode (pos) and 839 negative ion mode (neg). The chemical classification of these metabolites were mainly “organic acids and derivatives” (31.77%) and “lipids and lipid-like molecules” (23.59%) (Supplementary Figure S6). Multivariate statistical analysis of OPLS-DA under positive and negative ion modes showed that significant differences in metabolic patterns were existed between the CK and MC groups (Figure 4). Compared with the CK group, 103 DMs were identified under positive ion pattern, including 56 up-regulated and 47 down-regulated metabolites. The negative ion pattern identified 87 DMs, including 53 up-regulated and 34 down-regulated metabolites (Supplementary Figures S7–S9).

**Figure 5:**
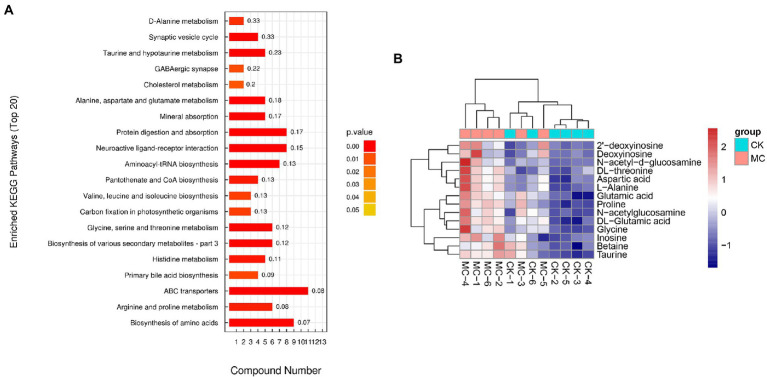
The pathways of differential metabolites in the intestinal of *L*. vannamei after MC-LR exposure. **(A)** The pathways of differential metabolites. **(B)** The differential metabolites of ABC transporters.

The metabolic pathways of the DMs were further analyzed (Figure 5A). The top five highly enriched pathways were mainly “ABC transporters” “biosynthesis of amino acids” “protein digestion and absorption” “neuroactive ligand-receptor interaction” “aminoacyl-tRNA biosynthesis” Additionally, many pathways of amino acid metabolism were also highly enriched, including “arginine and proline metabolism” “glycine, serine and threonine metabolism” “histidine metabolism” “alanine, aspartate and glutamate metabolism” and “taurine and hypotaurine metabolism” In the ABC transporters pathway, a total of 14 DMs were up-regulated, including dl-threonine, aspartic acid, l-alanine, glutamic acid, proline, dl-glutamic acid, glycine, taurine, betaine, N-acetyl-d-glucosamine, N-acetyl-glucosamine, 2′-deoxyinosine, deoxyinosine, and inosine (Figure 5B).

### Identification of intestinal metabolic markers

Certain organism health-related DMs were further identified (Table 1). For example, 7 carbohydrates abundance were increased, including pyruvate, muramic acid, N-acetyl-d-glucosamine, N-acetylglucosamine, N-acetyl-d-mannosamine, N-Acetylmannosamine, and dl-lactate. Fifteen amino acids abundance were altered, such as glycine, dl-threonine, dl-glutamic acid, d-pyroglutamic acid, proline, aspartic acid, l-alanine, l-methionine, glutamic acid, sarcosine, taurine, ergothioneine, γ-aminobutyric acid (GABA), and betaine were up-regulated; while γ-glutamylvaline was down-regulated. Five lipids such as linoleic acid, dodecanoic acid, myristic acid, taurocholate, and taurodeoxycholic acid were decreased. Additionally, 1 vitamin such as pyridoxine was decreased.

**Table 1:** Significantly altered metabolites in the intestines of *L. vannamei* after MC-LR exposure.

Name	VIP	Fold change	*p*-value	Categories
Pyruvate	1.58	3.11	0.01	Carbohydrates
Muramic acid	1.37	2.65	0.02	Carbohydrates
dl-Lactate	10.39	1.60	0.00	Carbohydrates
N-Acetyl-d-glucosamine	7.70	2.44	0.01	Carbohydrates
N-Acetylglucosamine	3.28	1.89	0.00	Carbohydrates
N-Acetyl-d-mannosamine	4.65	2.39	0.01	Carbohydrates
N-Acetylmannosamine	1.46	2.34	0.01	Carbohydrates
Glycine	2.16	1.70	0.01	Amino acids
dl-Threonine	1.47	1.28	0.04	Amino acids
dl-Glutamic acid	3.03	1.22	0.00	Amino acids
d-Pyroglutamic acid	1.38	1.15	0.01	Amino acids
Proline	7.52	1.52	0.00	Amino acids
Aspartic acid	3.08	1.51	0.01	Amino acids
l-Alanine	1.06	1.50	0.01	Amino acids
l-Methionine	3.61	1.49	0.04	Amino acids
Glutamic acid	5.36	1.36	0.00	Amino acids
Sarcosine	2.11	1.28	0.03	Amino acids
Taurine	7.26	1.22	0.05	Amino acids
Ergothioneine	5.18	3.58	0.00	Amino acids
γ-Aminobutyric acid	2.37	2.24	0.04	Amino acids
Betaine	22.06	1.15	0.05	Amino acids
γ-Glutamylvaline	7.31	0.86	0.00	Amino acids
Dodecanoic acid	4.38	0.50	0.00	Lipids
Linoleic acid	20.48	0.45	0.04	Lipids
Taurocholate	2.26	0.34	0.00	Lipids
Taurodeoxycholic acid	4.48	0.27	0.01	Lipids
Myristic acid	11.52	0.34	0.00	Lipids
Pyridoxine	2.66	0.27	0.00	Vitamins

### Integrated analysis of intestinal microbiota and metabolic phenotype

**Figure 6:**
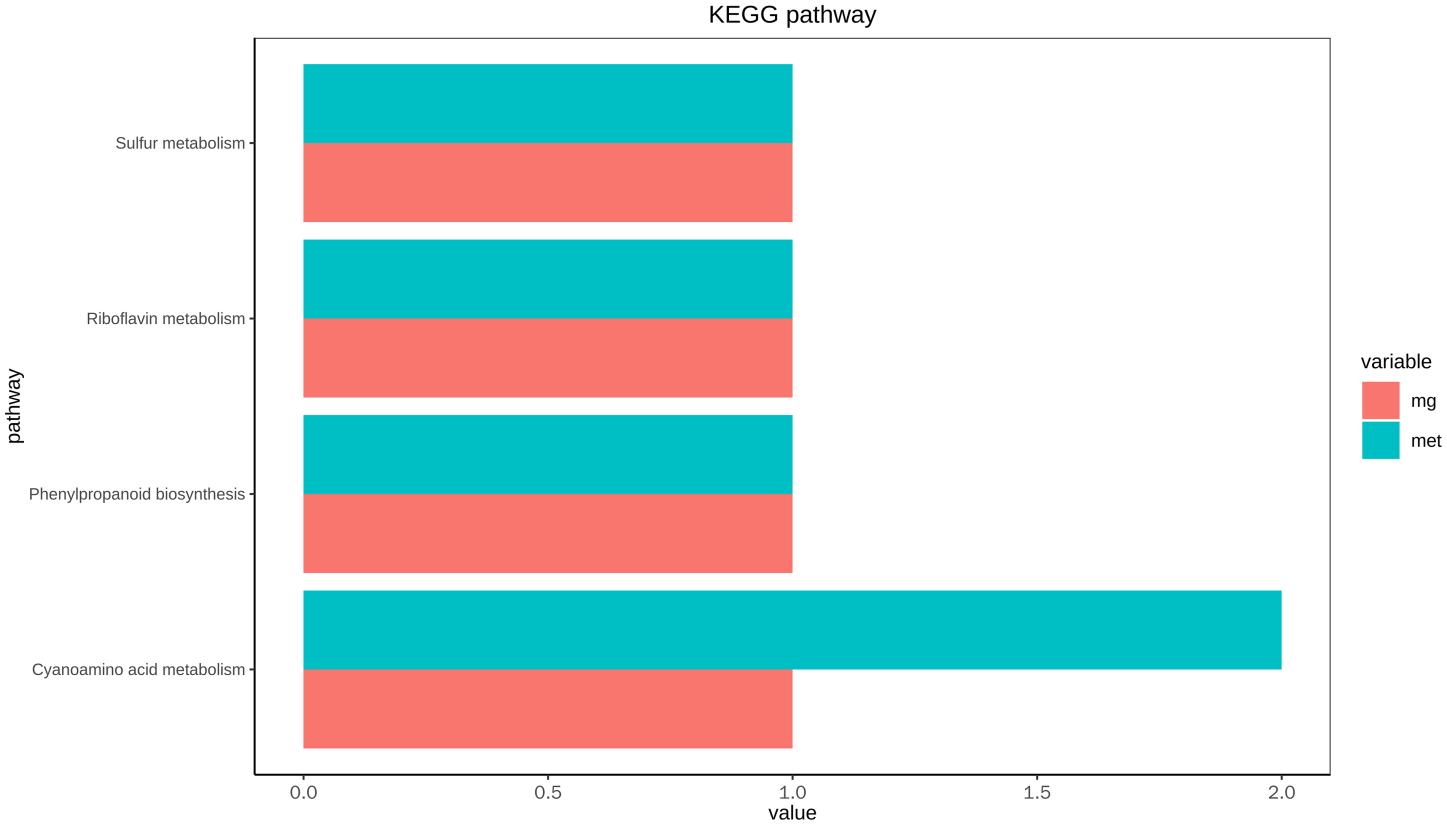
Correlation analysis of KEGG pathway of intestinal microbial metagenome and metabolome of *L. vannamei*.

The KEGG pathway of functional genes and metabolites with significant differences in abundance was statistically analyzed (Figure 6). The results showed that phenylpropanoid biosynthesis, and riboflavin, sulfur and cyanoamino acid metabolism were the high abundance pathways of intestinal microbial metabolism.

**Figure 7:**
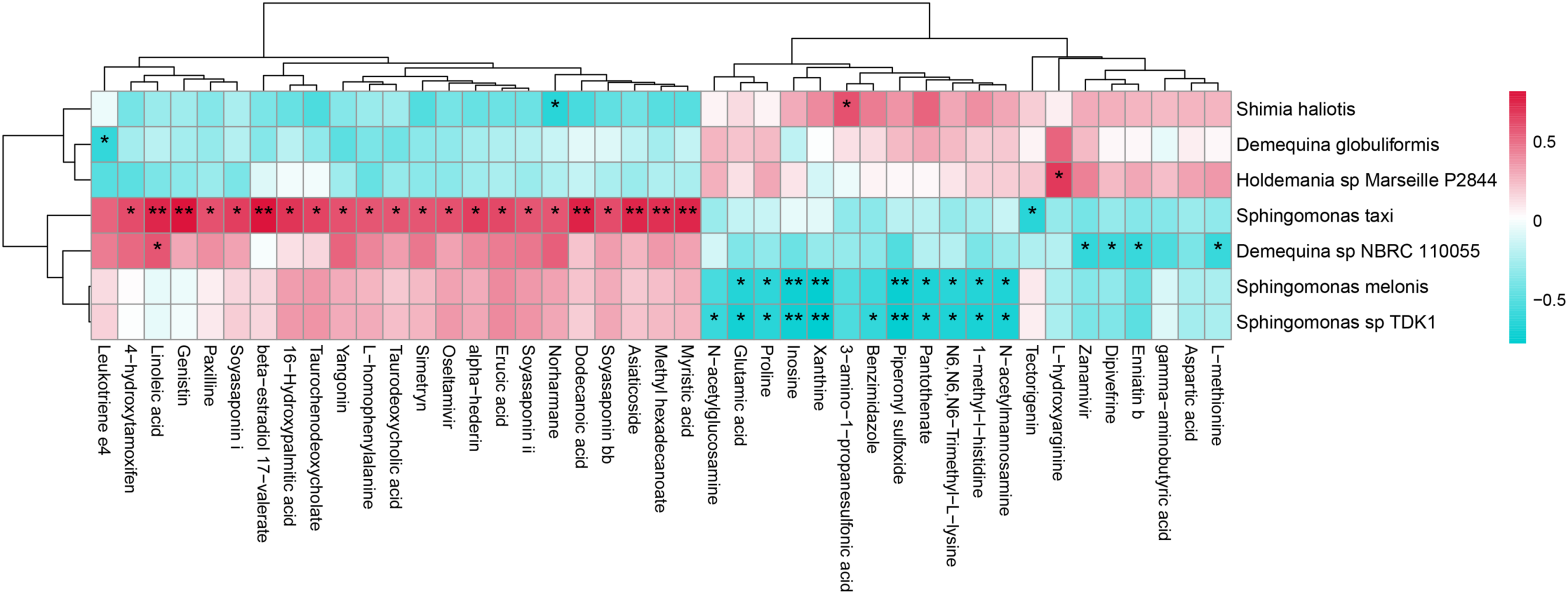
The correlation analysis between intestinal bacterial species and metabolites of *L. vannamei*. * represents a significant difference (*p < 0.05; **p < 0.01).

The correlation between intestinal bacterial species and metabolites was further analyzed (Figure 7). For instance, *D.* sp. *NBRC 110055* was positively correlated with linoleic acid, but negatively correlated with enniatin B and l-methionine. *Sphingomonas melonis* was negatively correlated with pantothenate and proline. *S. taxi* was positively correlated with α-hederin, asiaticoside, dodecanoic acid, linoleic acid, myristic acid, paxilline, soyasaponins, taurochenodeoxycholate, taurodeoxycholic acid, and yangonin. *Sphingomonas* sp. *TDK1* was negatively correlated with glutamic acid, proline and pantothenate. *Sphingomonas haliotis* was negatively correlated with norharmane.

**Figure 8:**
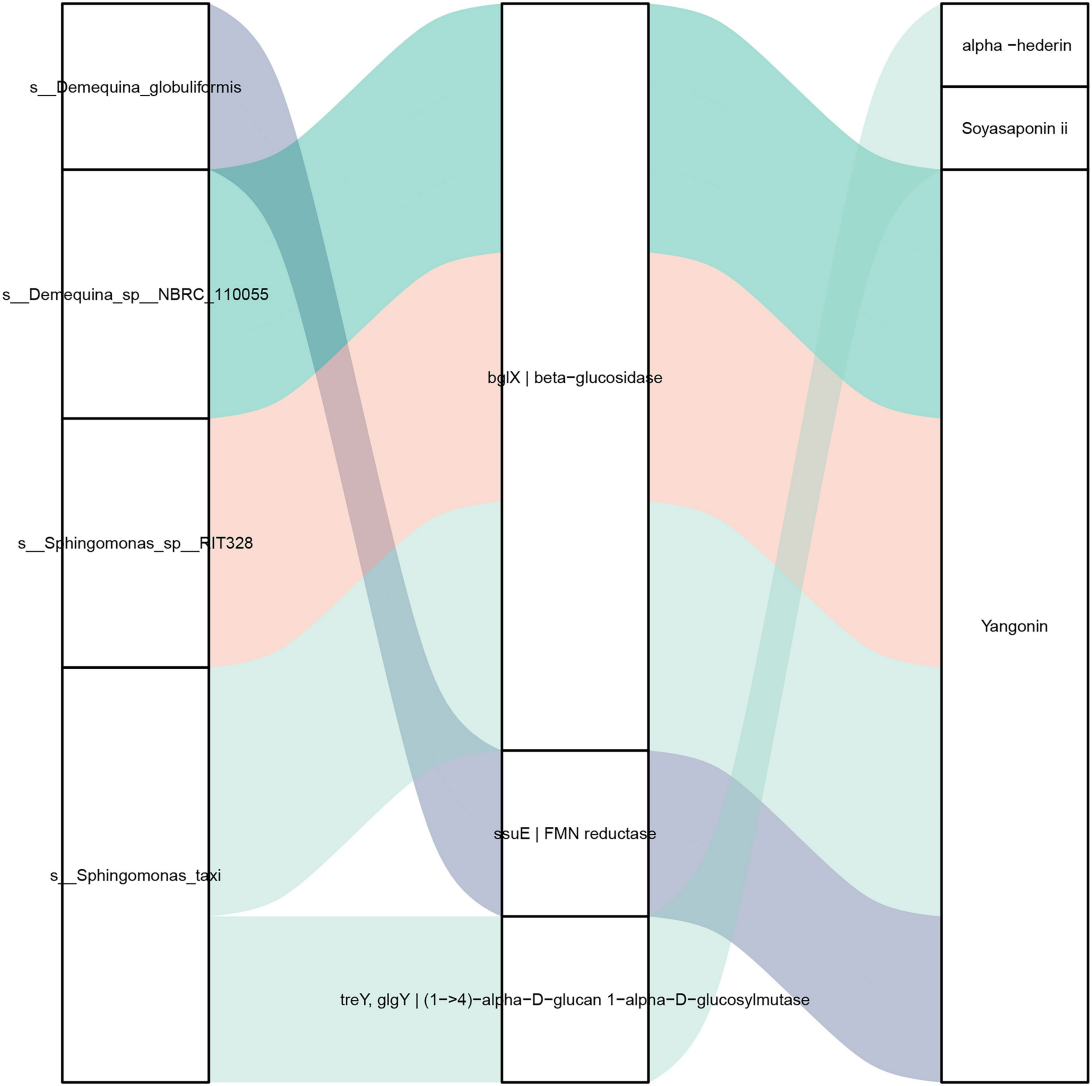
Correlation analysis of intestinal bacteria species, functional genes and metabolites of *L. vannamei*. Left: bacteria species, middle: functional genes, right: metabolites. The line between the bacteria species and functional gene indicates the contributing bacteria of the functional gene. The line between the functional genes and metabolites represents they have significant correlation (|*r*| > 0.8, *p* < 0.05).

The correlation between intestinal bacterial species, functional genes and metabolites was further analyzed (Figure 8). The bacteria *D.* sp. *NBRC 110055*, *S. taxi* and *S.* sp. *RIT328* were correlated with the metabolite yangonin through β-glucosidase (bglX) gene, while *D. globuliformis* was correlated with yangonin through flavin mononucleotide reductase (ssuE) gene. The bacteria *S. taxi* was correlated with the metabolites α-hederin and soyasaponin ii through (1 → 4)-α-d-glucan 1-α-d-glucosylmutase (treY) gene.

## Discussion

### Intestinal microbial metagenomic response to MC-LR exposure

The intestinal microbiota interacts with its host, thereby influencing many of the host physiological processes, and changes in their functions can affect host physiological homeostasis (Valdes et al., 2018; Holt et al., 2021). In this study, based on the functional annotations of eggNOG and KEGG, the gene functions of the intestinal microbial of *L. vannamei* were mainly amino acid and carbohydrate metabolism, which was consistent with the results of Gao et al. (2019). After MC-LR exposure, the functions of ABC transporter and “KO_EC 1.5.1.38” of intestinal microbial were increased, and “KO_EC 1.5.1.38” was mainly involved in sulfur and riboflavin metabolism. Microbial sulfur metabolism produces hydrogen sulfide (H_2_S), which is associated with colorectal cancer (Nguyen et al., 2021). Riboflavin, also known as vitamin B2, has antioxidant activity and is mainly involved in biological oxidation and energy metabolism *in vivo* (Ashoori and Saedisomeolia, 2014; Salman and Naseem, 2015). ABC transporters are membrane-binding proteins that participate in transmembrane transport of endogenous syncytic and xenobiotic compounds, contributing to the detoxification and excretion of toxic substances (Higgins, 1992; Wang et al., 2021). Therefore, it can be concluded that MC-LR exposure can induce the risk of H_2_S generation in intestinal microbial metabolism of *L. vannamei*, and the organism can improve antioxidant function and promote detoxification metabolism by enhancing ABC transporter and riboflavin metabolism.

Intestinal microbes can produce a large number of carbohydrate metabolism enzymes to influence digestion and metabolism of the host (Valdes et al., 2018). CAZymes encoded by intestinal microbes can catalyze the decomposition of glycoconjugates, oligosaccharides and polysaccharides into monosaccharides, and can also metabolize dietary fibers to produce short-chain fatty acids to promote the intestinal health of the host (Kaoutari et al., 2013). In this study, based on CAZy functional annotation, carbohydrate metabolism enzymes of intestinal microbial of *L. vannamei* were mainly glycoside hydrolase (GH) and glycosyltransferase (GT). After MC-LR exposure, the levels of α-mannosidase, glucan 1,3-β-glucosidase, glucan 1,4-β-glucosidase, coniferin β-glucosidase, β-N-acetylhexosaminidase, β-N-acetylglucosaminide phosphorylases, xylan 1,4-β-xylosidase, and exo-1,3-1,4-glucanase of intestinal microbial of the shrimp were increased, indicating that intestinal microbes respond to MC-LR stress by enhancing the capacity of carbohydrate hydrolysis.

Several potentially bacteria species exhibited variation after MC-LR exposure. *Demequina* is involved in starch degradation to glucose and dextrine (Al-naamani et al., 2015; Peruzzi et al., 2017). In this study, the relative abundances of three *Demequina* species (*D. flava*, *D.* sp. *NBRC 110055* and *D. sediminicola*) were increased in response to MC-LR exposure, suggesting that these bacteria may facilitate carbohydrate degradation. *P. damselae* is an opportunistic pathogen, which has high pathogenicity and can cause photobacteriosis of shrimp (Song et al., 1993; Vaseeharan et al., 2007). *S. marina* was originally isolated from the biofilm of a coastal fish farm (Choi and Cho, 2006), and may have pathogenicity related to coral white diseases based on metagenomic studies (Godwin et al., 2012; Séré et al., 2013). In this study, the relative abundances of *P. damselae* and *S. marina* were decreased in the MC group, indicating MC-LR exposure might affect the abundance of potentially harmful bacteria in the shrimp intestines.

### Intestinal metabolomics response to MC-LR exposure

Previous studies have shown that MC-LR exposure induces hepatopancreas metabolic phenotype and lipid homeostasis in *L. vannamei*, such as citric acid cycle as well as amino acid, linoleic acid, and arachidonic acid metabolism. However, intestinal metabolic characteristics of *L. vannamei* under MC-LR stress are still unclear. In this study, untargeted metabonomics further revealed that MC-LR exposure caused significant fluctuations in the intestinal metabolites in *L. vannamei*. Interestingly, the differential metabolites were mainly enriched in the ABC transporters, protein digestion and absorption, and amino acid biosynthesis and metabolism. These phenomena indicate that amino acid metabolism is the main toxic mechanism of MC-LR in *L. vannamei*, and the metabolic response of intestinal and hepatopancreas to MC-LR stress is different.

Amino acid metabolism can provide nutrients and participate in physiological processes in organisms (Eswarappa and Fox, 2013). ABC transporter can realize trans-membrane transport of various substances and has the detoxification function (Higgins, 1992; Wang et al., 2021). In this study, the up-regulation of amino acids also participated in ABC transporters. Hence, we speculated that the shrimp intestines might utilize ABC transporters and amino acid metabolism for adapting MC-LR stress, and the up-regulated amino acids could also compensate for the host’s requirements for amino acids. Pyridoxine (VB6) is a coenzyme component involved in the non-oxidative degradation of amino acids (Giri et al., 1997). In this study, the decreased level of pyridoxine further suggested that MC-LR influenced the homeostasis of amino acid metabolism in the shrimp intestines.

Lipid metabolism participates in fatty acid and energy sources for organisms (Lee et al., 2018). In this study, the decreased levels of linoleic acid, dodecanoic acid, myristic acid, taurocholate and taurodeoxycholic acid indicated that MC-LR affected intestinal lipid metabolism homeostasis. Taurocholate is considered to be a more efficient bile acid in fat absorption (Chesney et al., 1998). Taurine is involved in fat digestion as a bile acid conjugator (Kim et al., 2007). In this study, the decreased levels of taurocholate and taurodeoxycholic acid, and the increased level of taurine indicated that MC-LR might also affect the bile acid metabolism of the shrimp.

Additionally, several intestinal metabolites related to the health of the organism were also fluctuated after the shrimp exposed to MC-LR. For example, four amino acids including ergothioneine, betaine, GABA and taurine were all up-regulated. Ergothioneine is a natural antioxidant (Hartman, 1990). Betaine is a common feed additive that functions in anti-inflammatory, liver disease treatment, and intestinal health improvement (Santos et al., 2019; Huang et al., 2021). GABA is an neurotransmitter produced by microorganisms that enhances stress resistance in shrimp (Xie et al., 2015). Taurine can reduce inflammation and oxidative stress, and regulate blood sugar, lipids and energy metabolism by binding bile acids (Guizoni et al., 2020). In this study, the increased levels of ergothioneine, betaine, GABA and taurine indicated that the shrimp intestines might response positively to stress by producing these beneficial metabolites.

### Integration of metagenomic and metabolomic of intestinal microbial

Host-microbiota interactions can affect the immunity of aquatic animals (Li et al., 2019b; Xiao et al., 2021a,b). The integrated analysis of intestinal microbial metagenome and metabolome is conducive to elucidate the relationship between intestinal microbe and metabolites, thus identifying key biomarkers. In this study, intestinal bacteria species *D. globuliformis*, *D.* sp. *NBRC 110055*, *S. taxi* and *S.* sp. *RIT328* were correlated with the metabolites yangonin, α-hederin and soyasaponin ii. At present, there is a lack of study reports on these four bacteria species, their specific functions are still unclear and need to be further analyzed. α-hederin and soyasaponin have anti-tumor and inflammatory and immunomodulatory functions (Deng et al., 2019; Chitisankul et al., 2021). Yangonin has anti-cancer cell proliferation and antioxidant activity, which can also be used to treat liver diseases (Renchao et al., 2019). Thus speculate intestinal metabolites yangonin, α-hederin and soyasaponin ii might improve the resistance to MC-LR stress of *L. vannamei*, and can be used as potential functional metabolic markers for the development of biological agents. For these intestinal bacteria and metabolic markers identified in this study, their roles in the response of *L. vannamei* to MC-LR stress will be explored in the future, so as to lay the foundation for the research and development of biological agents.

## Conclusion

We investigated the effects of MC-LR on the intestinal microbial function of *L. vannamei* using an integrated metagenomic and metabolomic approach. MC-LR stress altered the gene functions of intestinal microbial, including ABC transporter, sulfur metabolism and riboflavin (VB2) metabolism, and induced a significant increase of eight carbohydrate metabolism enzymes. Alternatively, the metabolic pattern variation occurred the intestine, involving ABC transporters, protein digestion and absorption, and the biosynthesis and metabolism of amino acid; several functional metabolites including 7 carbohydrates, 14 amino acids, 5 lipids, and 1 vitamin were identified. Further, based on the integration of intestinal microbial metagenomic and metabolome, four bacteria species (*D. globuliformis*, *D.* sp. *NBRC 110055*, *S. taxi* and *S.* sp. *RIT328*) and three metabolites (yangonin, α-hederin and soyasaponin ii) biomarkers were identified. ABC transporter is an important target of intestinal microbiota of the shrimp in response to MC-LR stress. Overall, our study provides new insights into the effects of MC-LR on the intestinal microbial functions of *L. vannamei*.

## Data availability statement

The datasets presented in this study can be found in online repositories. The names of the repository/repositories and accession number (PRJNA862309) can be found in the article/Supplementary material.

## Ethics statement

The protocols for collecting and handling the animals in this study were approved by the Animal Care and Use Committee of the South China Sea Fisheries Research Institute, Chinese Academy of Fishery Sciences (SCSFRI-CAFS, No. nhdf2022-12).

## Author contributions

YD designed and performed the experiments, analyzed data, and wrote the manuscript. YX, SZ, and ZM contributed to sample collection and experimental analysis. XD, JZ, and YL assisted in resources, supervision, and project administration. All authors contributed to the article and approved the submitted version.

## Funding

This study was supported by National Natural Science Foundation of China (31902343); Guangdong Natural Science Foundation (2021A1515012084); Hainan Provincial Natural Science Foundation of China (322QN436); Guangzhou Basic and Applied Basic Research Project (202102080246); Young Scientific Talents Lifting Planning Project of Guangzhou Science and Technology Association (X20210201039); Agricultural Research Outstanding Talents Training Program (13210308); Central Public-interest Scientific Institution Basal Research Fund, South China Sea Fisheries Research Institute, CAFS (2022RC01 and 2021SD19); Central Public-interest Scientific Institution Basal Research Fund, CAFS (2021XT0604); Guangdong Provincial Special Fund for Modern Agriculture Industry Technology Innovation Teams (2019KJ141); and China-ASEAN Maritime Cooperation Fund, China-ASEAN Modern Marine Fishery Technical Cooperation, Industrialization Development and Demonstration.

## Conflict of interest

The authors declare that the research was conducted in the absence of any commercial or financial relationships that could be construed as a potential conflict of interest.

## Publisher’s note

All claims expressed in this article are solely those of the authors and do not necessarily represent those of their affiliated organizations, or those of the publisher, the editors and the reviewers. Any product that may be evaluated in this article, or claim that may be made by its manufacturer, is not guaranteed or endorsed by the publisher.

## Footnotes

^1^ http://www.kegg.jp/
