# Supplementary material for: Integration of metagenomic and metabolomic insights into the effects of microcystin-LR on intestinal microbiota of Litopenaeus vannamei
Source: Front Microbiol. 2022 Sep 23;13:994188. doi: 10.3389/fmicb.2022.994188 (PMC9537473; doi:10.3389/fmicb.2022.994188)
Supplement: Supplementary file 1 [file Data_Sheet_1.DOC]

**Supplementary materials**

**Integration of Metagenomic and Metabolomic Insights Into the Effects of Microcystin-LR on Intestinal Microbiota of *Litopenaeus vannamei***

Yafei Duan^1,2,3^, Yifu Xing^1^, Shimin Zeng^3^, Xueming Dan^3^, Zequan Mo^3^, Jiasong Zhang^1,2,*^, Yanwei Li^3,*^

^1^ *Key Laboratory of South China Sea Fishery Resources Exploitation & Utilization, Ministry of Agriculture and Rural Affairs, Guangdong Provincial Key Laboratory of Fishery Ecology and Environment, South China Sea Fisheries Research Institute, Chinese Academy of Fishery Sciences, Guangzhou 510300, PR China*

^2^ *Sanya Tropical Fisheries Research Institute, Sanya 572018, PR China*

^3^ *University Joint Laboratory of Guangdong Province, Hong Kong and Macao Region on Marine Bioresource Conservation and Exploitation, Guangdong Laboratory for Lingnan Modern Agriculture, College of Marine Sciences, South China Agricultural University, Guangzhou 510642, China*

### ***Correspondence:**

### Jiasong Zhang, jiasongzhang@hotmail.com

### Yanwei Li, yanweili@scau.edu.cn

**TABLE S1 Metagenomic sequencing data of intestinal microbial community of *L. vannamei***

| Sample | InsertSize(bp) | SeqStrategy | RawData | CleanData | Clean_Q20 | Clean_Q30 | Clean_GC(%) | Effective(%) | NonHostData |
| --- | --- | --- | --- | --- | --- | --- | --- | --- | --- |
| CK.1 | 350 | (150:150) | 6,153.21 | 5,945.72 | 89.44 | 82.47 | 41.95 | 96.628 | 3,083.08 |
| CK.2 | 350 | (150:150) | 6,165.06 | 6,040.92 | 89.95 | 82.75 | 42.41 | 97.986 | 2,082.10 |
| CK.3 | 350 | (150:150) | 6,371.64 | 6,279.62 | 92.59 | 85.91 | 40.75 | 98.556 | 4,220.92 |
| CK.4 | 350 | (150:150) | 6,832.78 | 6,728.67 | 91.56 | 85.13 | 43.76 | 98.476 | 2,956.20 |
| CK.5 | 350 | (150:150) | 6,284.32 | 6,258.49 | 94.51 | 87.67 | 48.48 | 99.589 | 5,758.20 |
| CK.6 | 350 | (150:150) | 6,560.25 | 6,431.94 | 91.28 | 84.45 | 41.55 | 98.044 | 3,025.56 |
| MC.1 | 350 | (150:150) | 6,388.22 | 6,261.71 | 89.35 | 81.97 | 43.59 | 98.02 | 2,293.18 |
| MC.2 | 350 | (150:150) | 6,298.06 | 6,179.74 | 88.98 | 81.62 | 44.06 | 98.121 | 2,107.48 |
| MC.3 | 350 | (150:150) | 6,322.71 | 6,233.48 | 91.42 | 84.77 | 43.86 | 98.589 | 3,258.20 |
| MC.4 | 350 | (150:150) | 6,639.55 | 6,576.58 | 93.56 | 87.85 | 46.4 | 99.052 | 4,455.28 |
| MC.5 | 350 | (150:150) | 6,052.53 | 6,037.13 | 96.77 | 91.84 | 44.27 | 99.745 | 5,634.08 |
| MC.6 | 350 | (150:150) | 6,654.72 | 6,626.73 | 96.13 | 91 | 45.55 | 99.579 | 5,900.40 |

**TABLE S2 Summary of intestinal microbial metagenomic data of *L. vannamei*.**

| Sample ID | Total len.(bp) | Num. | Average len. (bp) | N50 len. (bp) | N90 len. (bp) | Max len.(bp) |
| --- | --- | --- | --- | --- | --- | --- |
| CK.1 | 66,705,770 | 56,819 | 1,174.00 | 1,276 | 553 | 294,968 |
| CK.2 | 74,499,628 | 86,944 | 856.87 | 767 | 539 | 294,970 |
| CK.3 | 74,065,223 | 53,529 | 1,383.65 | 1,752 | 592 | 337,256 |
| CK.4 | 105,510,646 | 102,778 | 1,026.59 | 932 | 545 | 294,963 |
| CK.5 | 77,157,346 | 50,182 | 1,537.55 | 2,102 | 636 | 291,833 |
| CK.6 | 60,299,855 | 65,680 | 918.09 | 818 | 542 | 311,863 |
| MC.1 | 68,558,172 | 86,544 | 792.18 | 736 | 536 | 129,153 |
| MC.2 | 66,549,498 | 83,664 | 795.44 | 736 | 535 | 226,758 |
| MC.3 | 107,937,795 | 98,636 | 1,094.30 | 1,097 | 559 | 272,256 |
| MC.4 | 120,455,934 | 86,330 | 1,395.30 | 1,942 | 586 | 365,249 |
| MC.5 | 112,993,791 | 73,739 | 1,532.35 | 2,187 | 620 | 346,523 |
| MC.6 | 120,318,410 | 65,196 | 1,845.49 | 3,523 | 651 | 330,519 |

TABLE S3 Gene catalogue basic information summary

| Items | Number |
| --- | --- |
| ORFs NO. | 638019 |
| integrity:none | 27,283(4.28%) |
| integrity:all | 292,893(45.91%) |
| integrity:end | 197,397(30.94%) |
| integrity:start | 120,446(18.88%) |
| Total Len.(Mbp) | 258.72 |
| Average Len.(bp) | 405.5 |
| GC percent | 48.08 |


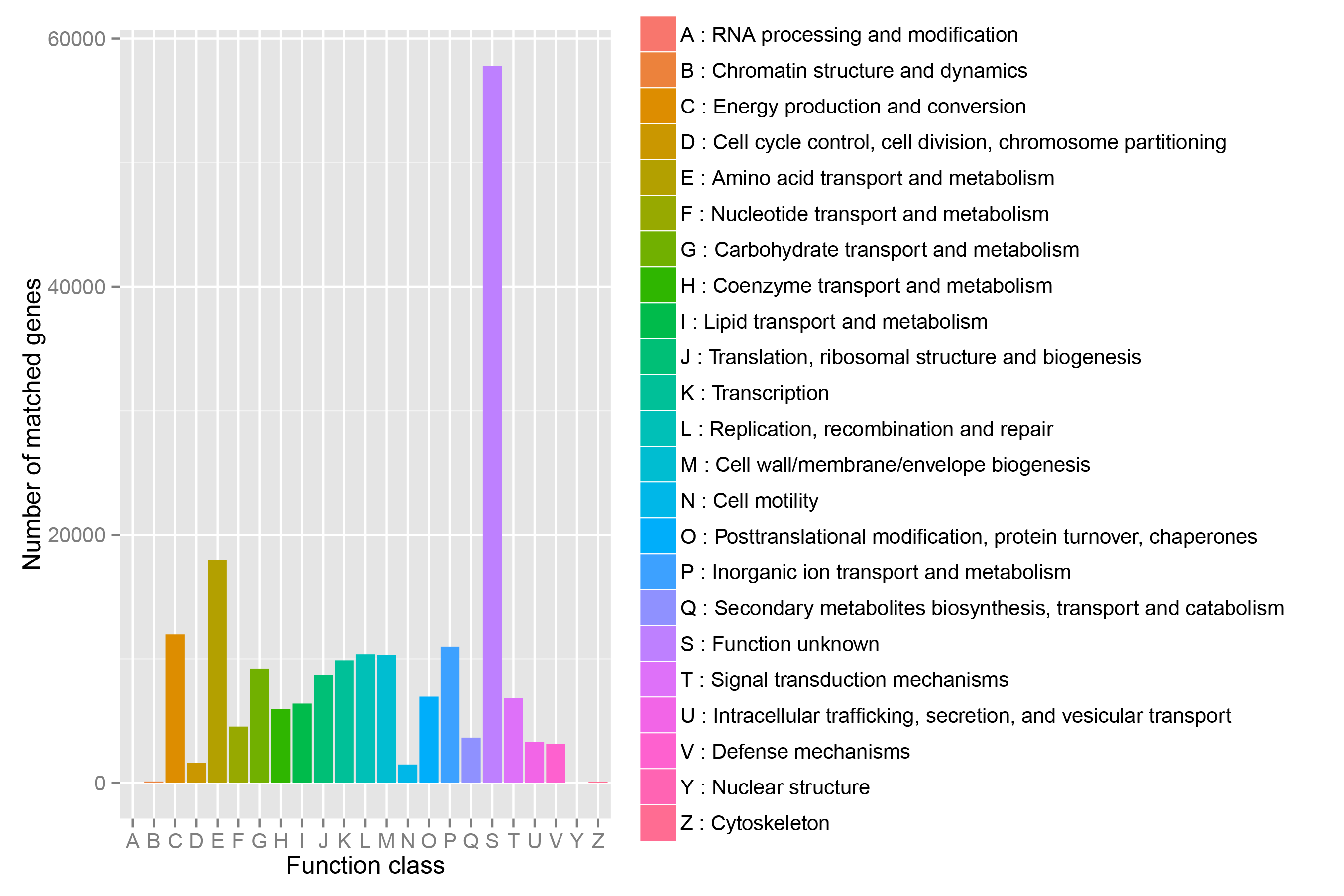


**FIGURE S1** The eggNOG functional annotation of intestinal microbial of *L. vannamei.*


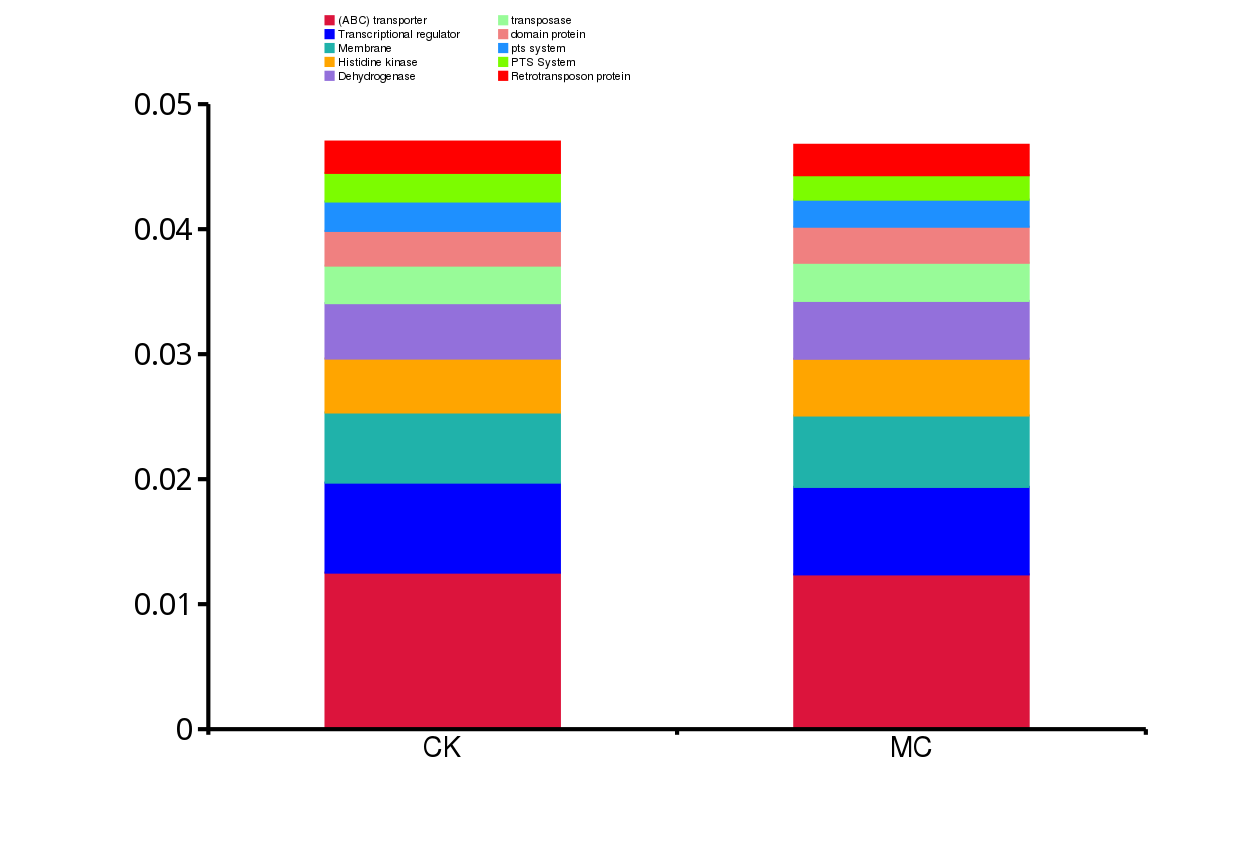


**FIGURE S2** Comparison of the eggNOG function of intestinal microbial of *L. vannamei* between the two groups (secondary level).


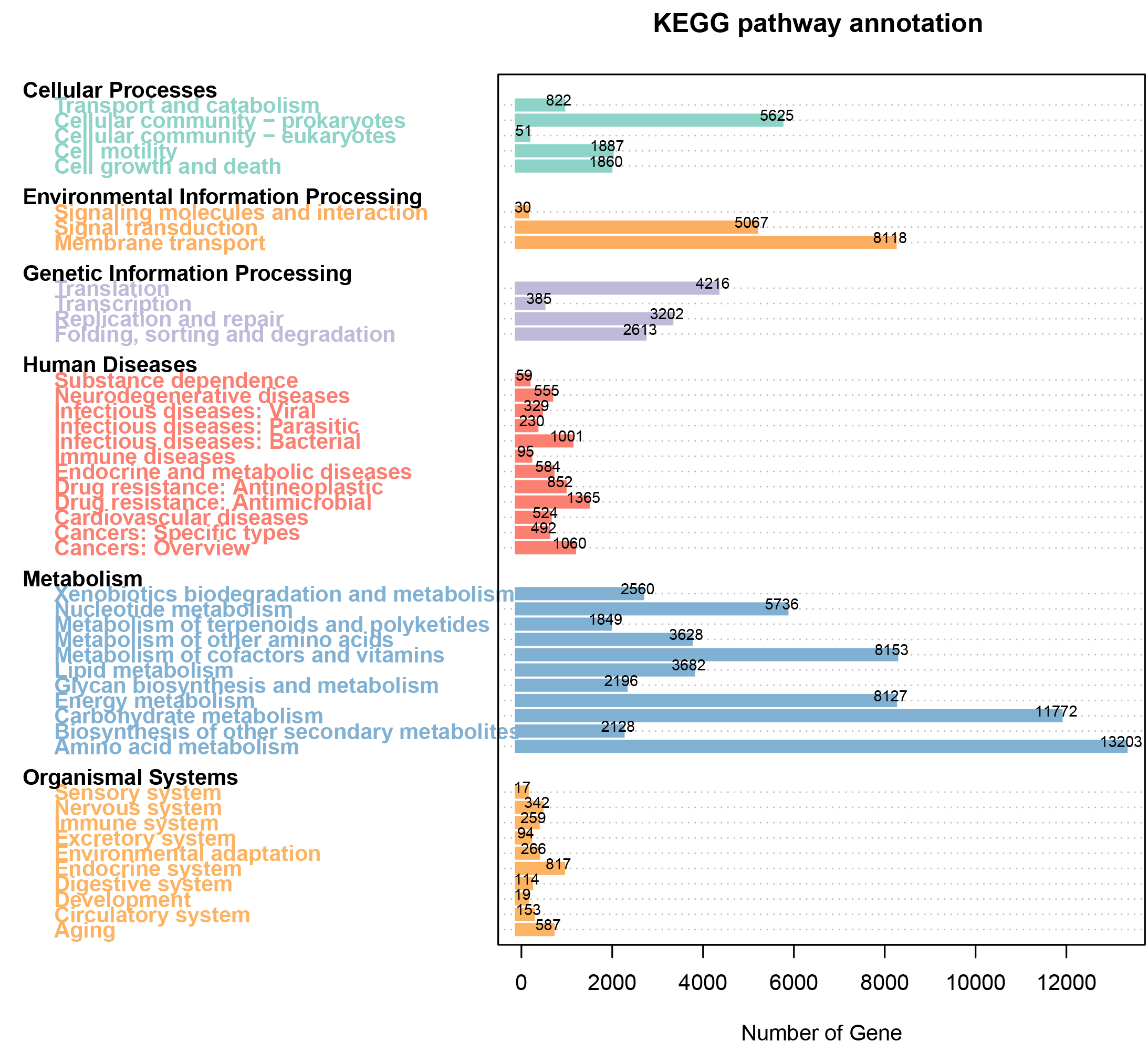


**FIGURE S3** The KEGG functional annotation of intestinal microbial of *L. vannamei.*


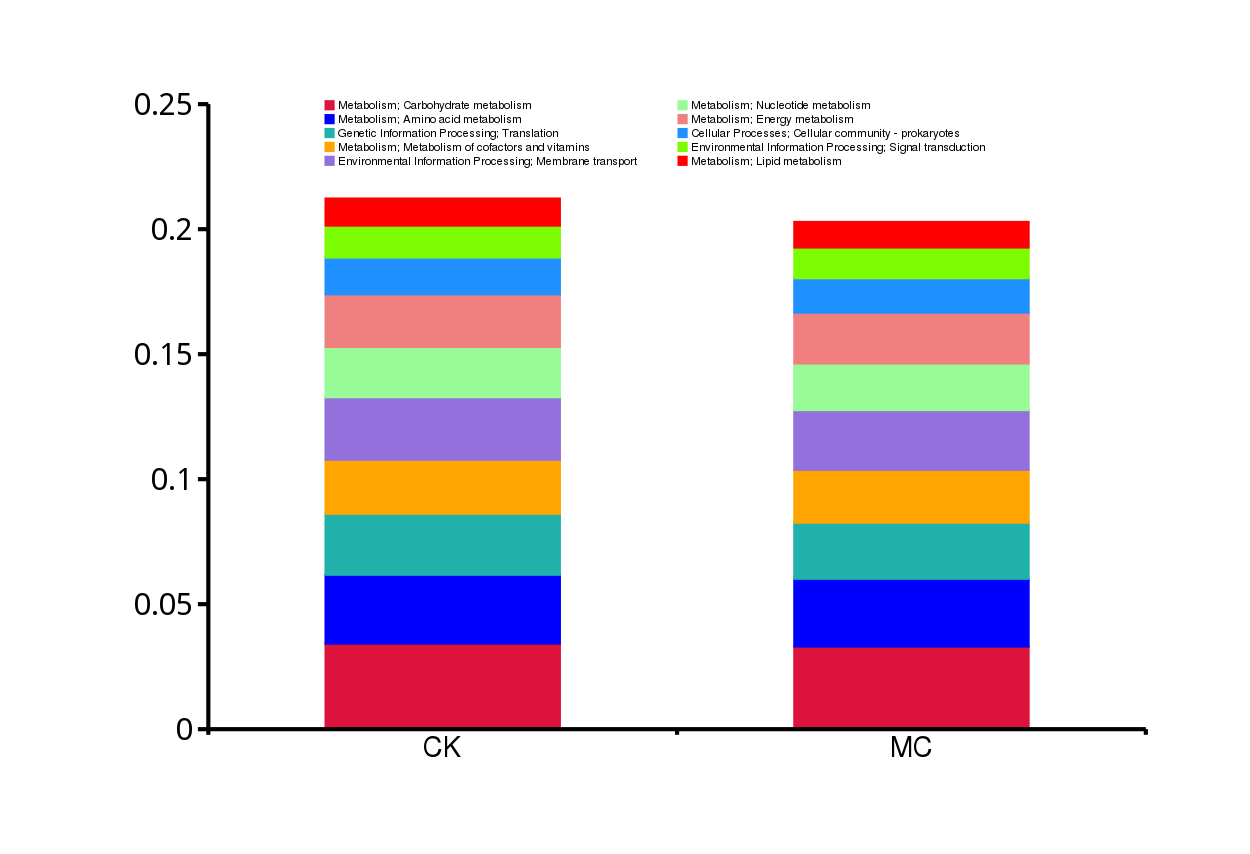


**FIGURE S4** Comparison of the KEGG function of intestinal microbial of *L. vannamei* between the two groups (secondary level).


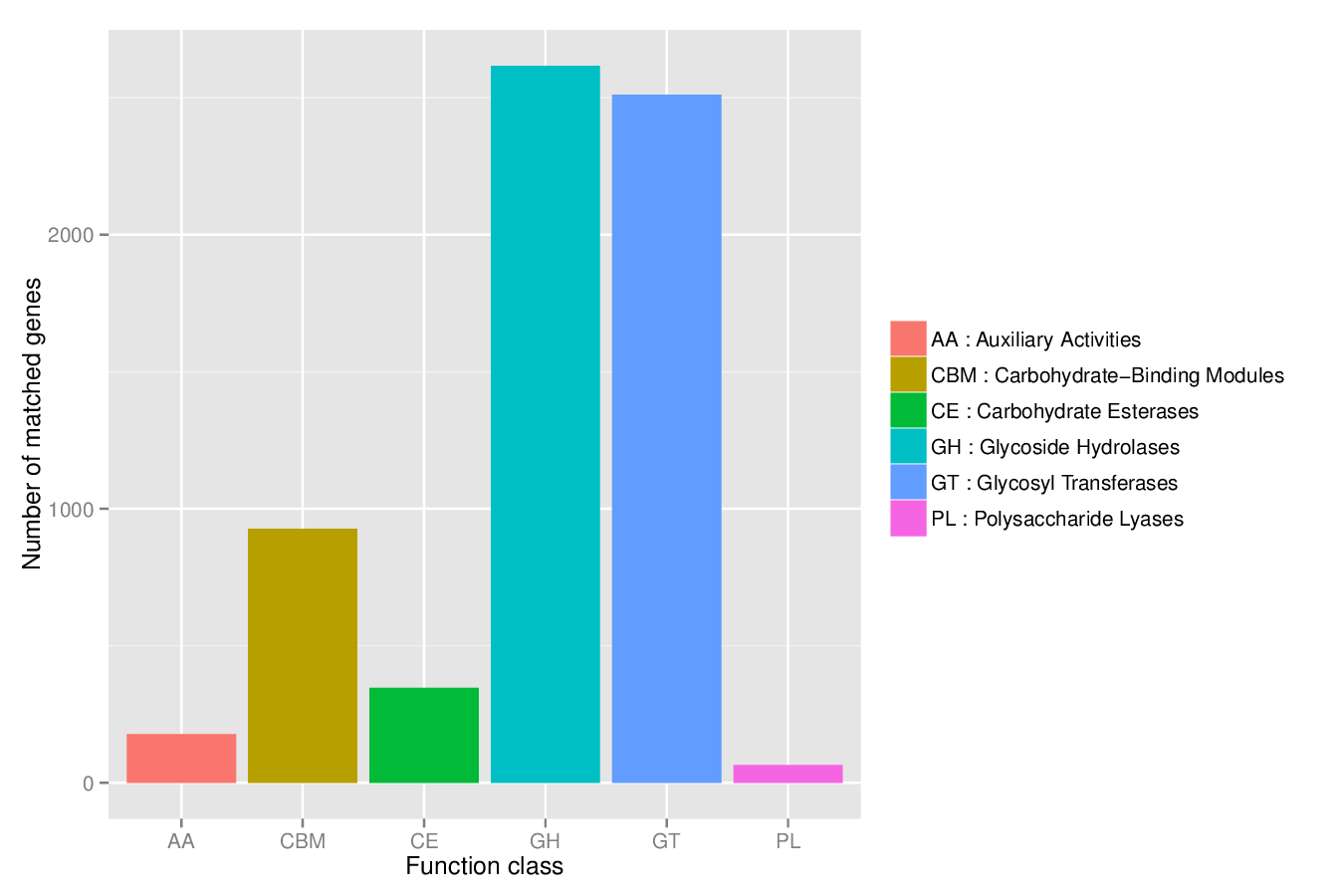


**FIGURE S5** The CAZy functional annotation of intestinal microbial of *L. vannamei.*


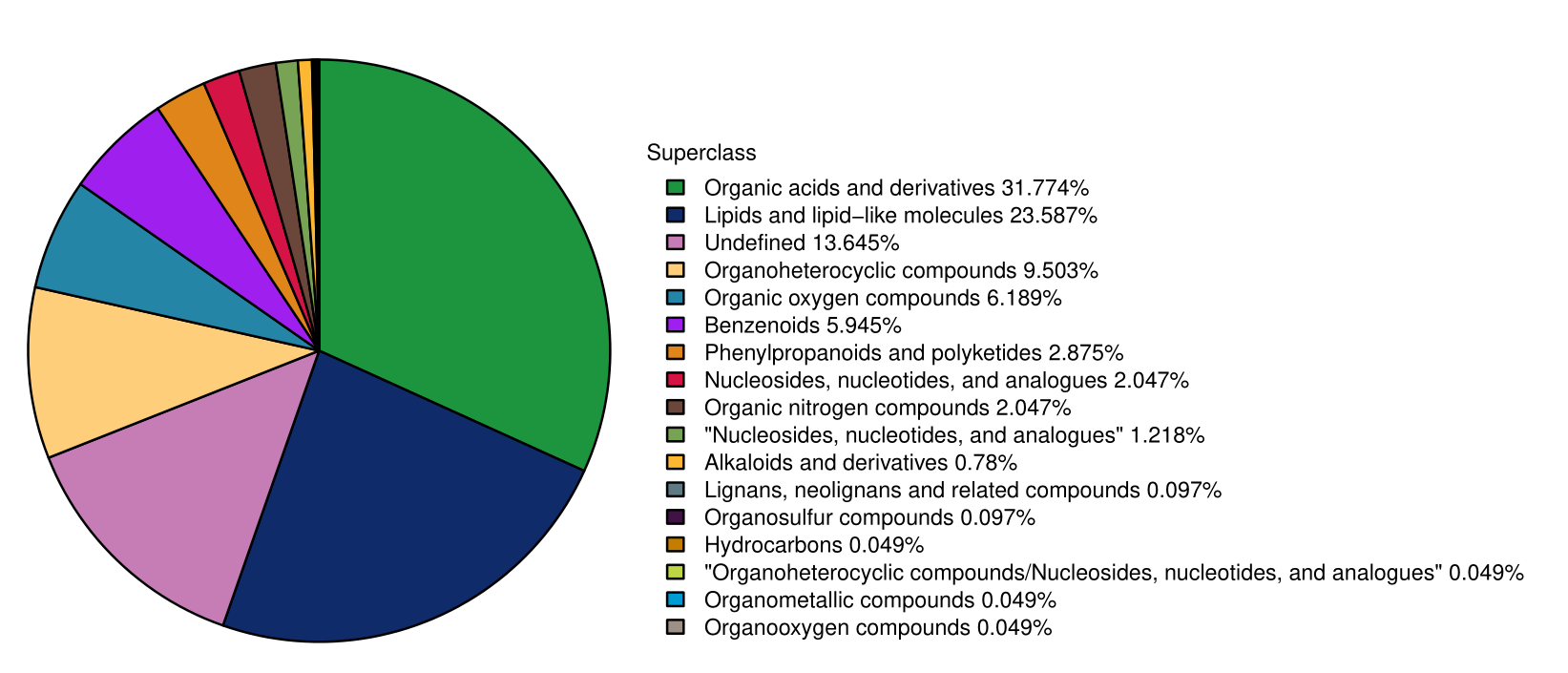


**FIGURE S6** Function classification of intestinal metabolites in *L. vannamei.*


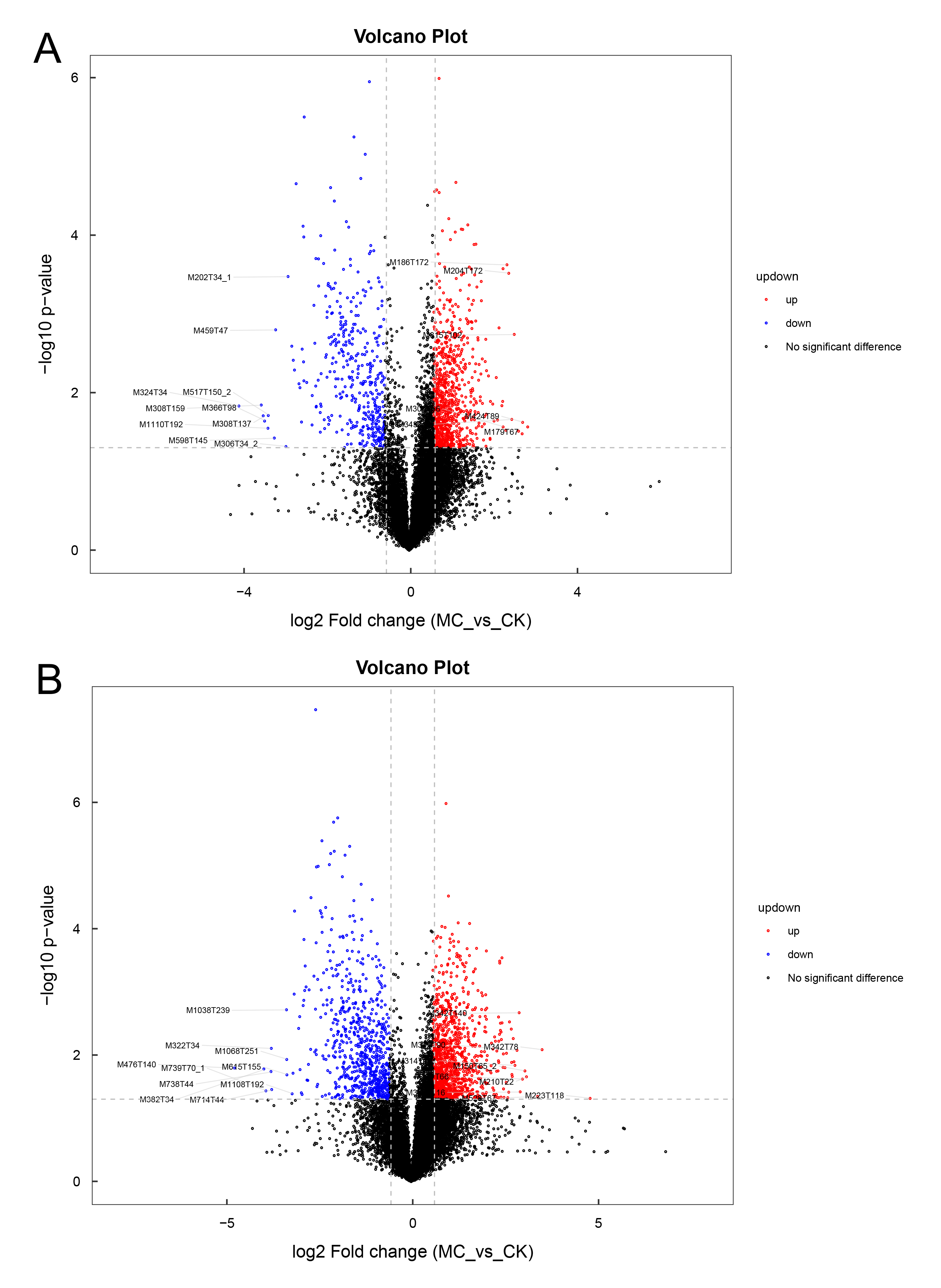


**FIGURE S7** Volcano plot of differential metabolites in intestinal of *L. vannamei.* (A) Positive ion mode. (B) Negative ion mode.


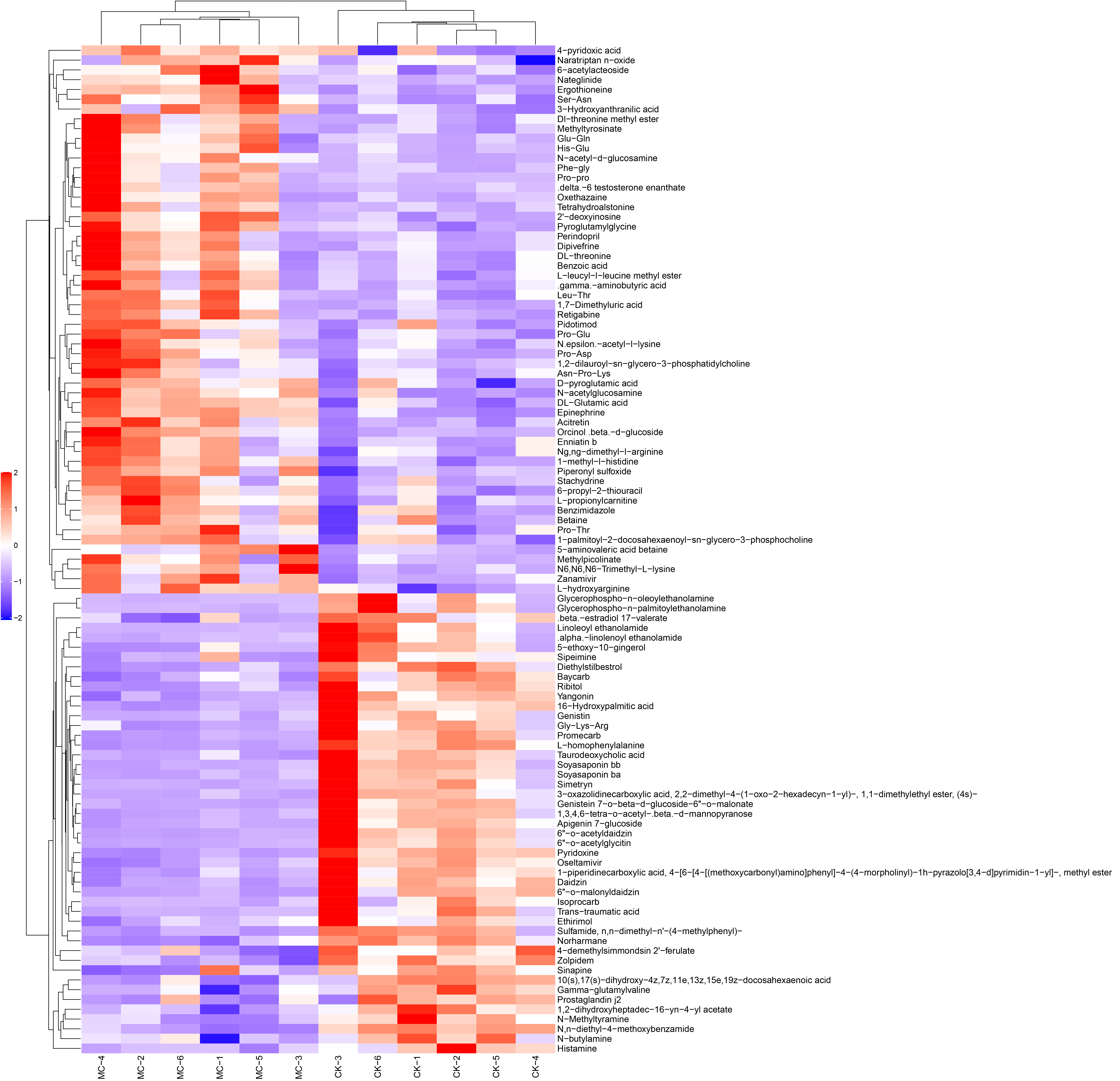


**FIGURE S8** Clustering of intestinal differential metabolites of *L. vannamei* in positive ion mode.


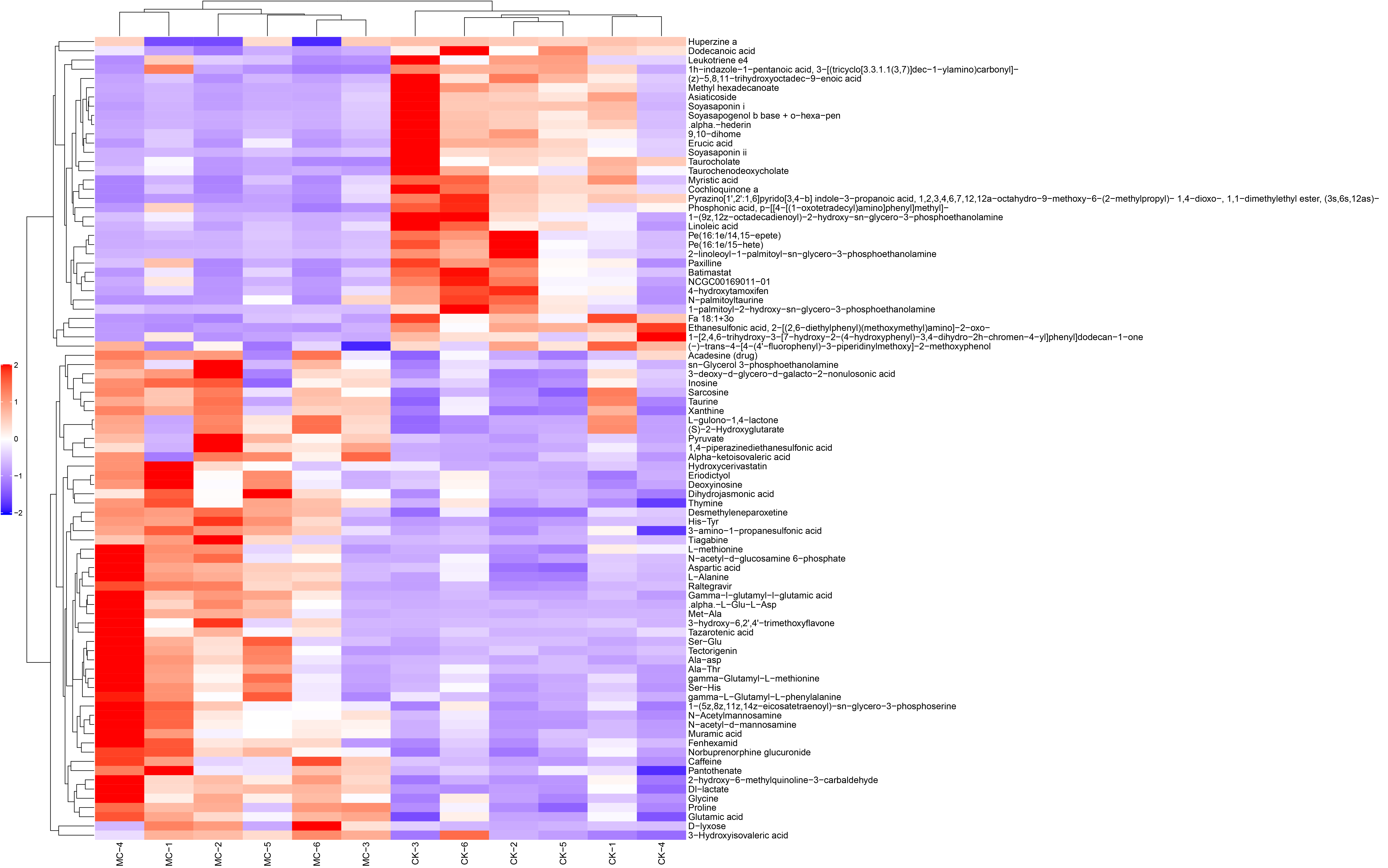


**FIGURE S9** Clustering of intestinal differential metabolites of *L. vannamei* in negative ion mode.
